# Diagnostic accuracy of a minimal immunohistochemical panel in at/rt molecular subtyping, correlated to dna-methylation profiling

**DOI:** 10.1186/s40478-023-01630-w

**Published:** 2023-08-21

**Authors:** Arnault Tauziède-Espariat, Julien Masliah-Planchon, Mamy Andrianteranagna, Philipp Sievers, Felix Sahm, Andreas von Deimling, Lauren Hasty, Olivier Delattre, Kévin Beccaria, Alice Métais, Fabrice Chrétien, Pascale Varlet, Franck Bourdeaut

**Affiliations:** 1 Department of Neuropathology, GHU Paris - Psychiatry and Neuroscience, Sainte-Anne Hospital, 1, rue Cabanis, Paris, 75014 France; 2 Institut de Psychiatrie et Neurosciences de Paris (IPNP), UMR S1266, INSERM, IMA-BRAIN, Paris, France; 3 Université de Paris Cité, Paris, France; 4 Laboratory of Somatic Genetics, Institut Curie, PMDT, Paris Sciences Lettres Research University, Paris, France; 5 Research In Pediatric, Adolescent and Young Adult Oncology, Laboratory of Translationnal Research in Pediatric Oncology, Institut Curie Institute, SIREDO Center Care, INSERM U830, Paris Sciences Lettres Research University, Innovation, Paris, France; 6 Department of Neuropathology, Institute of Pathology, University Hospital Heidelberg, Heidelberg, Germany; 7 Clinical Cooperation Unit Neuropathology, German Consortium for Translational Cancer Research (DKTK), German Cancer Research Center DKFZ, Heidelberg, Germany; 8 Department of Pediatric Neurosurgery, APHP, Necker Hospital, Université Paris Descartes, Sorbonne Paris Cite, Paris, 75015 France

**Keywords:** AT/RT, DNA-methylation profiling, TYR, SHH, MYC, Immunohistochemistry.

Atypical Teratoid/Rhabdoid Tumors (AT/RT) are malignant pediatric tumors of the Central Nervous System (CNS) and are molecularly characterized by a biallelic alteration of the *SMARCB1* (95%) or *SMARCA4* (5%) genes [1]. Transcriptional and DNA-methylation analyses can be used to classify these tumors into three distinct subgroups: SHH (overexpressing genes implicated in neurogenesis and NOTCH signaling, such as *ASCL1*, with additional sub-entities recently reported), TYR (overexpressing melanosomal marker genes such as *TYR, MITF*, and *OTX2*), and MYC (overexpressing *MYC* and *HOXC* clusters). These subgroups seem to be associated with distinct genetic and clinical features [1–3] but their significance in terms of prognosis is still unclear given the discrepant results reported in cohorts of patients treated with different therapies [1]. Thus, the prognosis and theranostic significance of these molecular subgroups still needs to be performed, which may be facilitated in near future prospective trials, and by using routine subgroup-specific biomarkers.

Our work studied a case series of 51 pediatric AT/RT (all *SMARCB1-*deficient) to evaluate the sensitivity/specificity of various combinations of immunohistochemical (IHC) markers to predict the molecular subgrouping. Using previously reported results and in-house datasets [3, 4], we retained Tyrosinase (Clone OCA1/812, 1:400, Abcam, Cambridge, United Kingdom), OTX2 (Clone 1H12C4B5, 1:600; Thermo Fisher, Rockford, USA), MITF (Clone D5, 1:100; Dako, Glostrup, Denmark), MYC (Clone Y69, 1:100, Abcam, Cambridge, United Kingdom), ASCL1 (polyclonal, 1:50; Sigma-Aldrich, Saint-Louis, USA), and SOX11 (Clone MRQ58, 1:100, Sigma-Aldrich, Saint-Louis, USA) as a potentially discriminating panel of markers. The DNA methylation analysis was performed with the Illumina EPIC 850k array, using the v12.5 of the Heidelberg classifier (https://www.molecularneuropathology.org/mnp/). Consistently, 42/51 were assigned to a subgroup of AT/RT with a calibrated score (≥ 0.9) (10 MYC, 24 SHH, and 8 TYR). The nine remaining cases were classified as AT/RT-TYR (n = 4), AT/RT-MYC (n = 4), and control tissue (n = 1) but with a low calibrated score and were thus excluded from subsequent analyses. Their overall distribution within subgroups is depicted by the specificity of t-Distributed Stochastic Neighbor Embedding (t-SNE) analysis (Supplementary Material 1: Fig. S1). Altogether, 42 well-defined AT/RT were considered for further comparison with immunohistochemical subtyping.

First, because of its low sensitivity (54% of correct subtyping), ASCL1 was excluded from our analyses. Next, nineteen different combinations of five different antibodies were tested to evaluate the accuracy of AT/RT subgrouping using the IHC markers we selected (Supplementary Material 2: Fig. S2). Panels 5 (SOX11-MYC-Tyrosinase), 15 (MYC-Tyrosinase), 16 (MYC-OTX2-MITF), 17 (MYC-OTX2-Tyrosinase), and 18 (MYC-MITF-Tyrosinase) presented the highest accuracy, in perfect concordance with the DNA-methylation profiling for 34/42 (81%) cases) (Supplementary Material 2: Fig. S2A and Supplementary Material 3: Table S1). Particularly, there was perfect concordance between IHC and DNA-methylation profiling for the TYR subgroup (100%). However, in 6/42 (14.3%) cases, IHC and DNA-methylation lead to discrepant conclusions (Fig. 1A-B), with the cases being labeled as belonging to the MYC subgroup by IHC and SHH by DNA-methylation (Supplementary Material 2: Fig. S2B and Table S1). Of note, RNA-sequencing analysis was available for five of these cases, which, consistently with IHC, all clustered with the AT/RT-MYC tumors (Fig. 1C). Finally, IHC failed to assign a subgroup in 2/42 cases (4.7%). Among the multi-marker panels, the best results were obtained with the combination of SOX11-MYC-Tyrosinase (panel 5), with a sensitivity and specificity to diagnose each methylation-defined subtype of: 71% and 100% (for SHH), 90% and 81% (for MYC), and 100% and 100% (for TYR). However, because a subset of AT/RT-MYC may express SOX11, IHC MYC has to be negative in the face of SOX11 positivity to suggest an AT/RT-SHH.

**Fig. 1:**
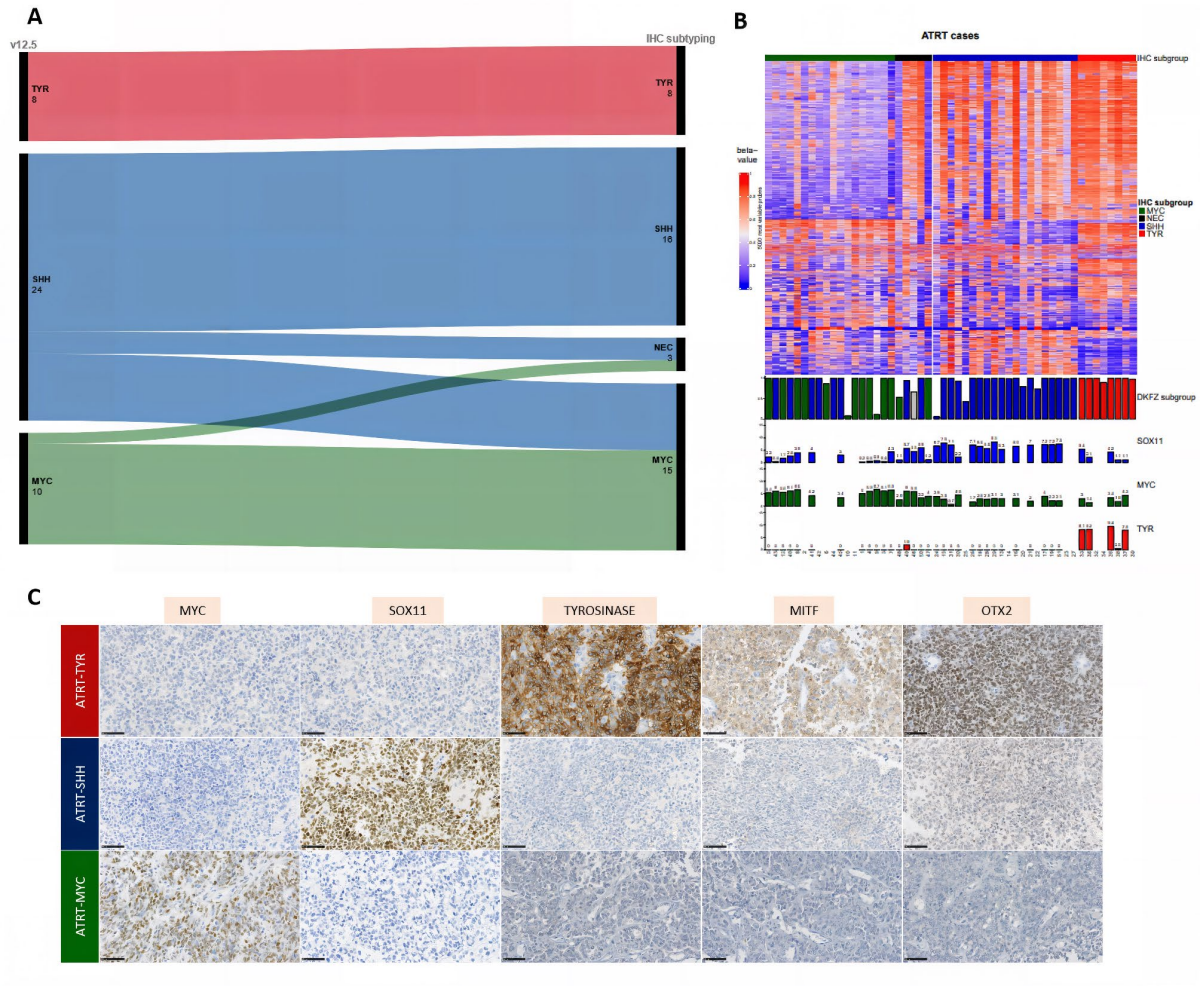
Comparison of molecular sub-typing by DNA-methylation analysis and immunohistochemistry. **A**: Alluvial diagram showing the assignment to subgroups by IHC subtyping using the panel 5 (left) and DNA-methylation profiling using the version v12.5 of the classifier. Cases with a calibrated score ≤ 0.9 were included in the “no match” group. **B**: Heatmap of DNA methylation beta-value using the top 5000 most variable probes. Samples are grouped according to the AT/RT subgroup predicted by our IHC method (top annotation). Unsupervised hierarchical clustering was applied to probes using Euclidean metric and Ward linkage. Sample subgroups identified using the DKFZ brain tumor classifier are plotted in the first layer of bottom annotation with the color indicating the AT/RT subgroup (red: TYR, blue: SHH and green: MYC). The height of the bar corresponds to the classification score (from 0 to 1). The three other layers of bottom annotation indicate respectively the expression level of *SOX11*, *MYC* and *TYR* genes in log2(TPM + 1) whenever RNA-seq data were available. **C**: A representative case with immunohistochemical findings for all subgroups (magnification x400). Black scale bars represent 50 μm Comparison of molecular sub-typing by DNA-methylation analysis and immunohistochemistry IHC: immunohistochemistry; NEC: Not Elsewhere Classified (the tumor was not classified in a subgroup)

This case series is the first to demonstrate the interest for the use of an antibody panel when sub-typing AT/RT. Our results confirm previously published outcomes showing that Tyrosinase expression is correlated to the AT/RT-TYR subgroup but may be encountered in a subset of SHH [5, 6]. OTX2 immunostaining is routinely used by neuropathologists in medulloblastoma subgrouping and represents a good candidate in the IHC panel for AT/RT subtyping [7]. We evidenced for the first time that SOX11 may constitute a good surrogate for the SHH subgroup whereas the sensitivity/specificity of ASCL1 was lowly informative in our series as reported elsewhere [3]. We also evidenced that a subset of AT/RT SHH - defined by methylation profiling - do express proteins that would be expected to relate them to another group based on RNA-expression data. The consistency between transcript and protein expressions suggests that the discrepancies between expression and methylation may reveal an actual diversity within the SHH group predicted by the current version of the classifier, with some AT/RT-SHH harboring markers predicted as being expressed in the MYC subgroup. Remarkably, the concordance between epigenetic and IHC subtyping is perfect for infratentorial AT/RT SHH (10/10 with a concordant sub-typing), but much less so for supratentorial AT/RT SHH (5/11 for cases with available data). This difference may have a potential biological significance and needs to be further explored.

To conclude, an immunostaining panel that includes MYC, SOX11 and Tyrosinase should be included in future clinical trials to study their clinicopathologic relevance and potential for use as surrogate markers.

### Electronic supplementary material

Below is the link to the electronic supplementary material.

Supplementary Material 1: Fig. S1. t-distributed stochastic neighbor embedding (t-SNE) analysis of the DNA methylation profiles of the 51 investigated tumors alongside selected reference samples of the DKFZ classifier (v12.5). Reference DNA methylation classes: AT/RT, *MYC* (Atypical teratoid/rhabdoid tumor, MYC-subtype); AT/RT, *SHH* (Atypical teratoid/rhabdoid tumor, SHH-subtype), AT/RT, *TYR* (Atypical teratoid/rhabdoid tumor, TYR-subtype), CNS NB, *FOXR2* (CNS neuroblastoma, FOXR2-activated), CNS tumor, *BCOR* ITD (CNS tumor with *BCOR* internal tandem duplication); CTRL, HEMI (Control tissue, cerebral hemisphere); ETMR, *C19MC* (Embryonal tumor with multilayered rosettes, C19MC-altered); ETMR, *DICER1* (Embryonal tumor with multilayered rosettes, DICER1-altered); MB, *non-WNT/ non-SHH* (Medulloblastoma, non-WNT, non-SHH); MB, *SHH* (Medulloblastoma, SHH-activated); MB, *WNT* (Medulloblastoma, WNT-activated). Cohort cases are designated by their number

Supplementary Material 2: Fig. S2. Additional immunohistochemical results.A: Comparison of results for molecular subtyping using nineteen different immunohistochemical panels (x: panels; y: number of cases). NEC: Not Elsewhere Classified. *designate the panels with the highest accuracy for subtyping. B: Discrepant cases with immunohistochemical findings (magnification x400). Black scale bars represent 50 μm

Supplementary Material 3: Detailed data of the cohort and synthesis

## Data Availability

Proteomic datasets were deposited to the Proteomics Identifications Database (PRIDE) with ac-cession number PXD016832.
