# Supplementary figures and images for: Diagnostic accuracy of a minimal immunohistochemical panel in at/rt molecular subtyping, correlated to dna-methylation profiling
Source: Acta Neuropathol Commun. 2023 Aug 21;11:136. doi: 10.1186/s40478-023-01630-w (PMC10440909; doi:10.1186/s40478-023-01630-w)

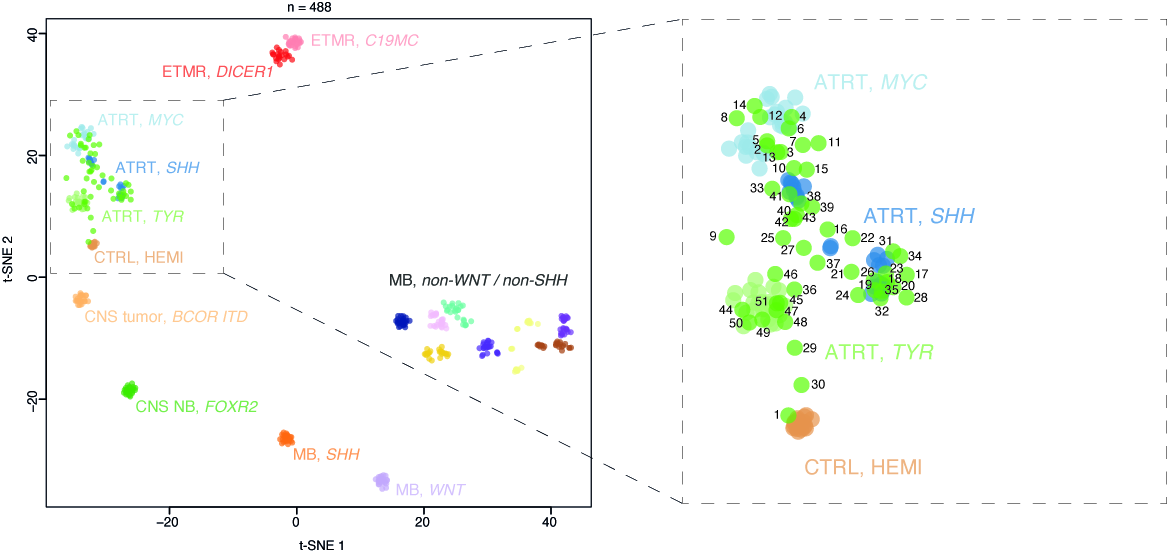

Supplement: Supplementary file 1 — Supplementary Material 1: Fig. S1. t-distributed stochastic neighbor embedding (t-SNE) analysis of the DNA methylation profiles of the 51 investigated tumors alongside selected reference samples of the DKFZ classifier (v12.5). Reference DNA methylation classes: AT/RT, MYC (Atypical teratoid/rhabdoid tumor, MYC-subtype); AT/RT, SHH (Atypical teratoid/rhabdoid tumor, SHH-subtype), AT/RT, TYR (Atypical teratoid/rhabdoid tumor, TYR-subtype), CNS NB, FOXR2 (CNS neuroblastoma, FOXR2-activated), CNS tumor, BCOR ITD (CNS tumor with BCOR internal tandem duplication); CTRL, HEMI (Control tissue, cerebral hemisphere); ETMR, C19MC (Embryonal tumor with multilayered rosettes, C19MC-altered); ETMR, DICER1 (Embryonal tumor with multilayered rosettes, DICER1-altered); MB, non-WNT/ non-SHH (Medulloblastoma, non-WNT, non-SHH); MB, SHH (Medulloblastoma, SHH-activated); MB, WNT (Medulloblastoma, WNT-activated). Cohort cases are designated by their number [file 40478_2023_1630_MOESM1_ESM.tif]

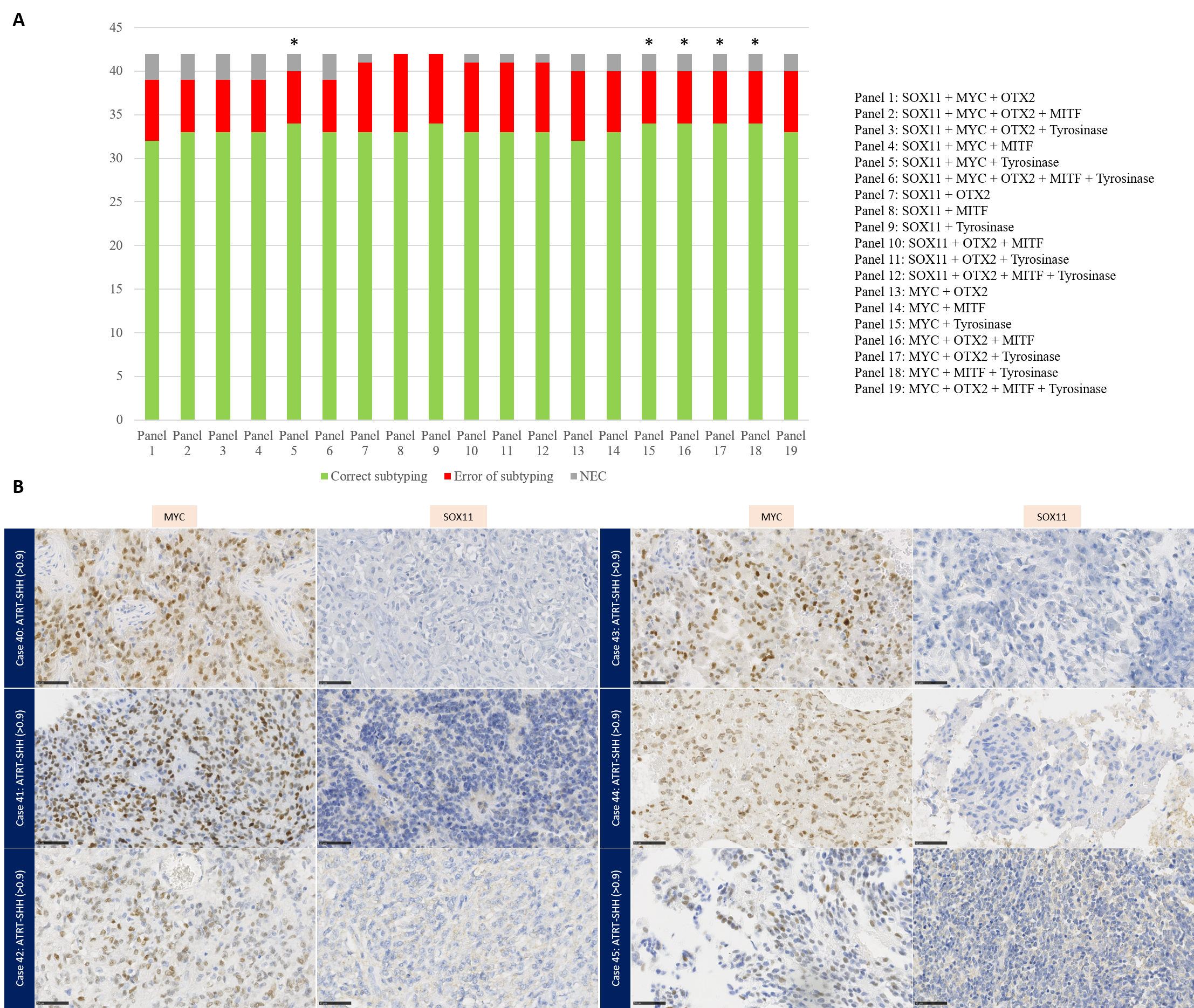

Supplement: Supplementary file 2 — Supplementary Material 2: Fig. S2. Additional immunohistochemical results.A: Comparison of results for molecular subtyping using nineteen different immunohistochemical panels (x: panels; y: number of cases). NEC: Not Elsewhere Classified. *designate the panels with the highest accuracy for subtyping. B: Discrepant cases with immunohistochemical findings (magnification x400). Black scale bars represent 50 μm [file 40478_2023_1630_MOESM2_ESM.tif]
